# Supplementary material for: RápidoPGS: a rapid polygenic score calculator for summary GWAS data without a test dataset
Source: Bioinformatics. 2021 Jun 19;37(23):4444–50. doi: 10.1093/bioinformatics/btab456 (PMC8652106; doi:10.1093/bioinformatics/btab456)
Supplement: btab456_Supplementary_Data [file btab456_supplementary_data.zip › Supplementary_note.pdf]

Supplementary Note

Guillermo Reales, Elena Vigorito, Martin Kelemen, and Chris Wallace

Assume we have a trait

$$Y = \sum_{i=1}^{m} X_i \beta_i + \epsilon$$

with $\beta_i \sim N(0, W)$ and the sum taken over m SNPs that are causal for the trait. Heritability is

$$h^2 = \frac{\operatorname{Var}(Y - \epsilon)}{\operatorname{Var} Y} \Rightarrow \operatorname{Var} \epsilon = (1 - h^2) \operatorname{Var}(Y)$$

Then it follows that

$$\begin{aligned}
\operatorname{Var}(Y) &= \operatorname{Var}(\sum_{i=1}^{m} X_i \beta_i) + \operatorname{Var}(\epsilon) \\
&= \sum_{i=1}^{m} \beta_i^2 \operatorname{Var}(X_i) + (1 - h^2) \operatorname{Var}(Y)
\end{aligned}$$

Here Var is taken across individuals, and β_i is fixed but unknown for each SNP i. We replace β_i^2 by its expected value across SNPs, W. $\sum \operatorname{Var}(X_i)$ is also unknown, but we have estimates of $\operatorname{Var}(\hat{\beta}_i) = \frac{\operatorname{Var} Y}{N_i \operatorname{Var} X_i}$ where N_i is the number of individuals with data at SNP i. Therefore

$$\sum_{i=1}^{m} \operatorname{Var}(X_i) \simeq \sum_{i=1}^{m} \frac{\operatorname{Var} Y}{N_i \operatorname{Var}(\hat{\beta}_i)} = \operatorname{Var} Y \sum_{i=1}^{m} \frac{1}{N_i \operatorname{Var}(\hat{\beta}_i)}$$

where the sum is taken across all m causal SNPs. We do not know what the causal SNPs are, but assume instead that $N_i, \operatorname{Var} \hat{\beta}_i$ take the same distribution across causal SNPs $1, \ldots, m$ and non-causal SNPs $m + 1, \ldots, p$. Then we can estimate

$$\sum_{i=1}^{m} \frac{1}{N_i \operatorname{Var} \hat{\beta}_i} \simeq \frac{m}{p} \sum_{i=1}^{p} \frac{1}{N_i \operatorname{Var} \hat{\beta}_i}$$

Finally, m is still unknown, but we have already specified the fraction of truly causal SNPs we expect as π so we estimate $\widehat{(\frac{m}{p})} = \pi$. Thus, putting it all together, we can write

$$\begin{aligned}
\operatorname{Var}(Y) &= W \sum_{i=1}^{m} \operatorname{Var}(X_i) + (1 - h^2) \operatorname{Var}(Y) \\
&\simeq W \operatorname{Var}(Y) \pi \sum_{i=1}^{p} \frac{1}{N_i \operatorname{Var} \hat{\beta}_i} + (1 - h^2) \operatorname{Var}(Y)
\end{aligned}$$

so that

$$W \simeq h^2 \bigg/ \pi \sum_{i=1}^{p} \frac{1}{N_i \operatorname{Var} \beta_i}$$
