# Supplementary figures and images for: RápidoPGS: a rapid polygenic score calculator for summary GWAS data without a test dataset
Source: Bioinformatics. 2021 Jun 19;37(23):4444–50. doi: 10.1093/bioinformatics/btab456 (PMC8652106; doi:10.1093/bioinformatics/btab456)

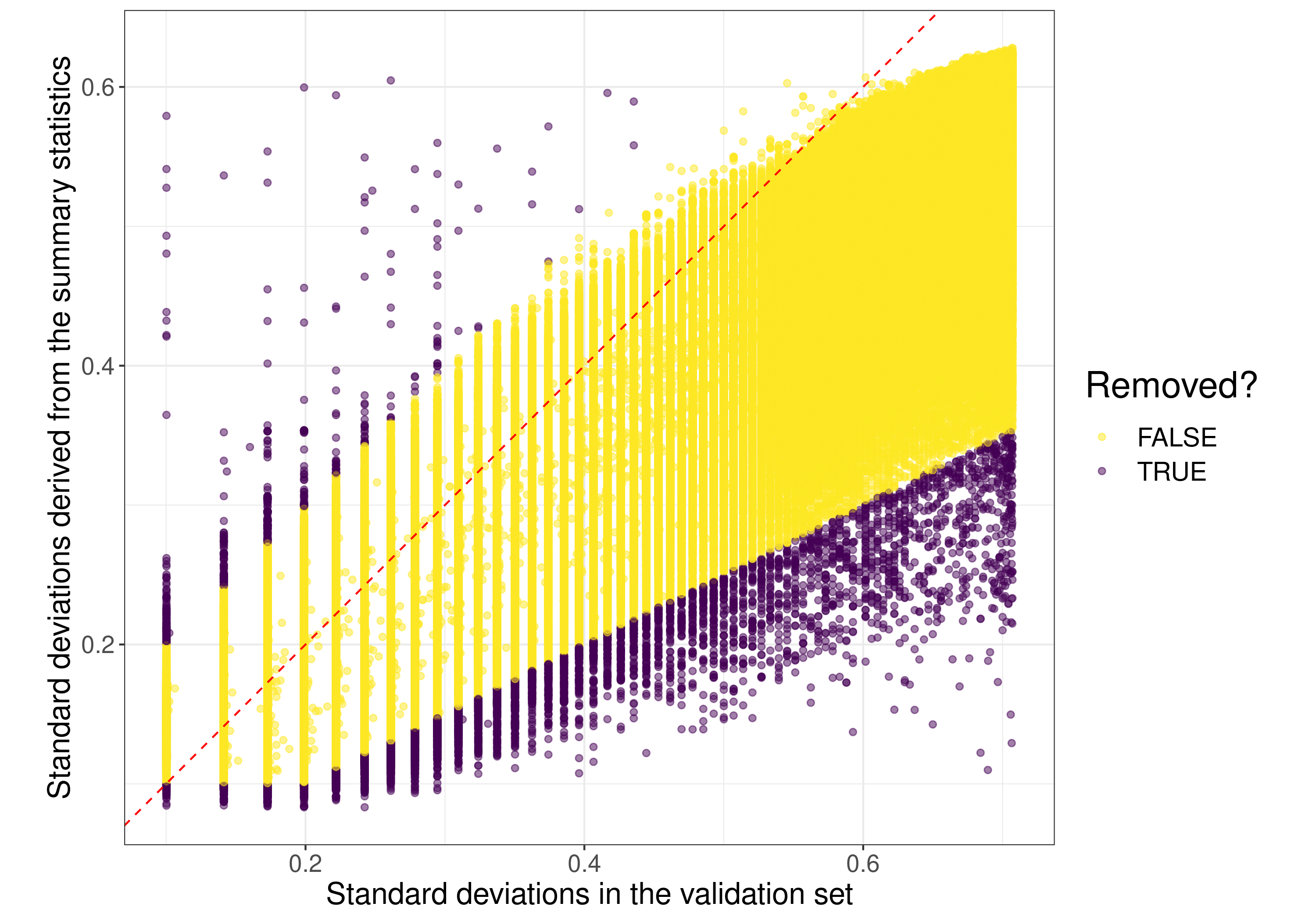

Supplement: btab456_Supplementary_Data [file btab456_supplementary_data.zip › FigS1_Asthma.png]

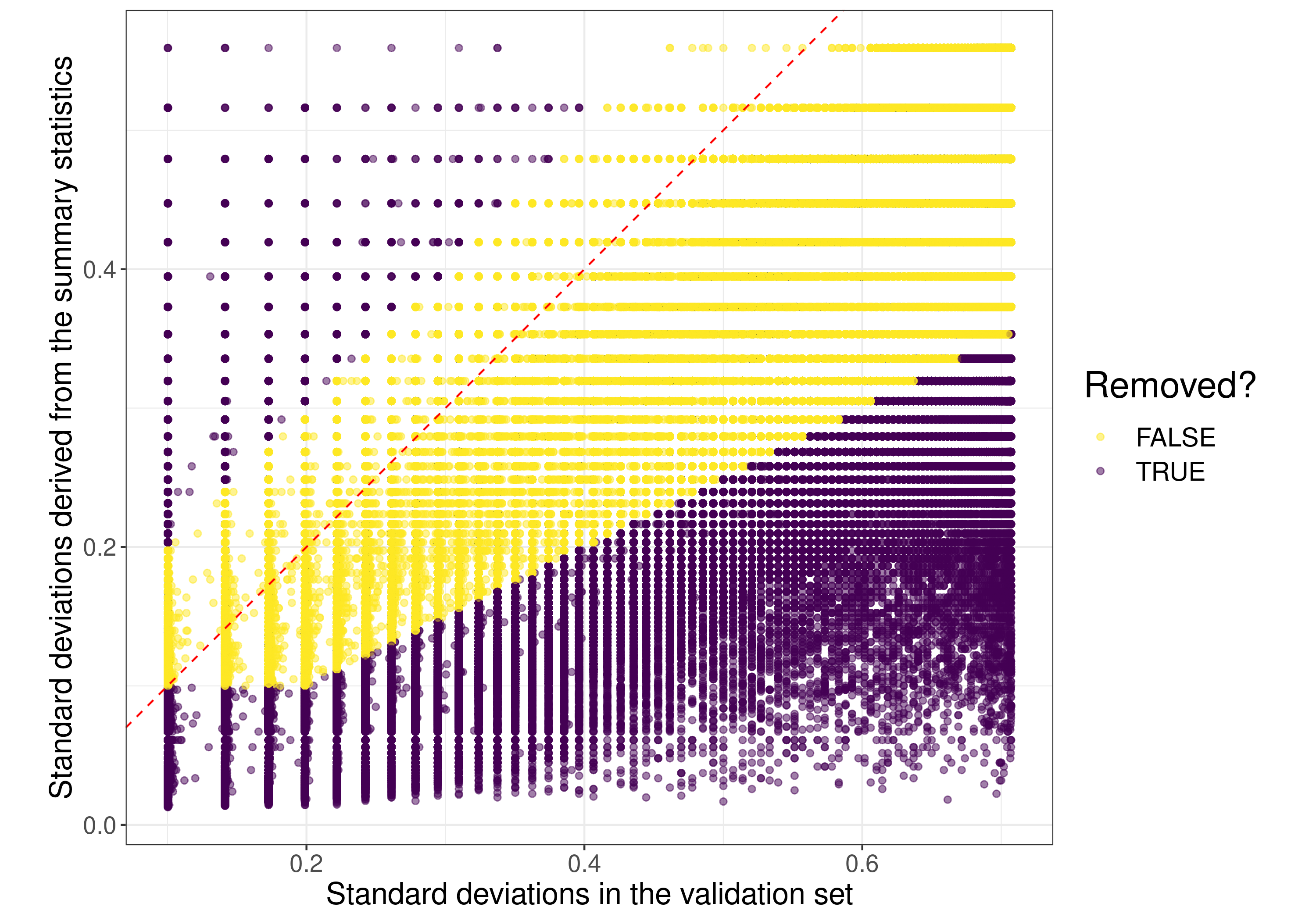

Supplement: btab456_Supplementary_Data [file btab456_supplementary_data.zip › FigS10_T2D.png]

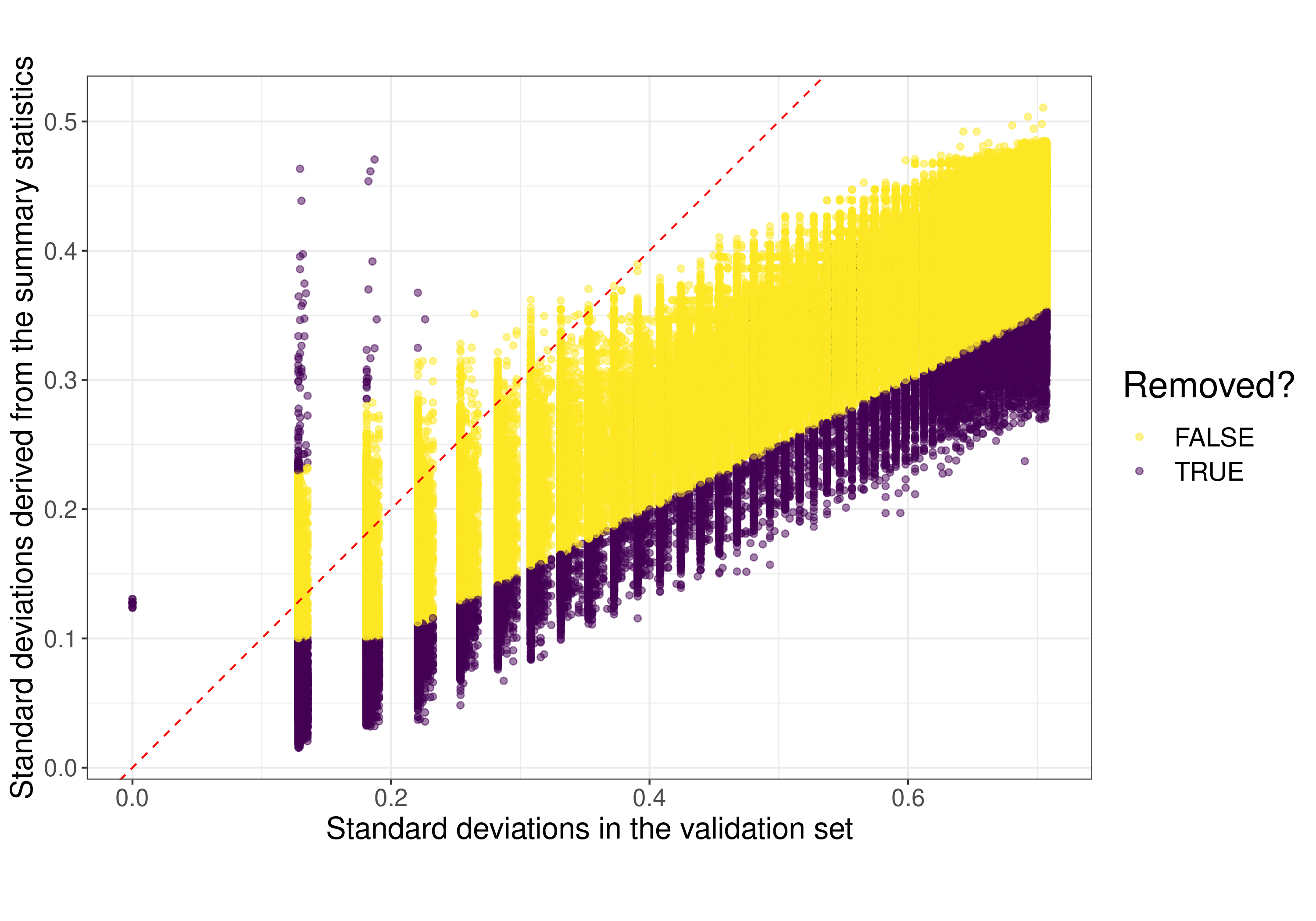

Supplement: btab456_Supplementary_Data [file btab456_supplementary_data.zip › FigS2_BMI.png]

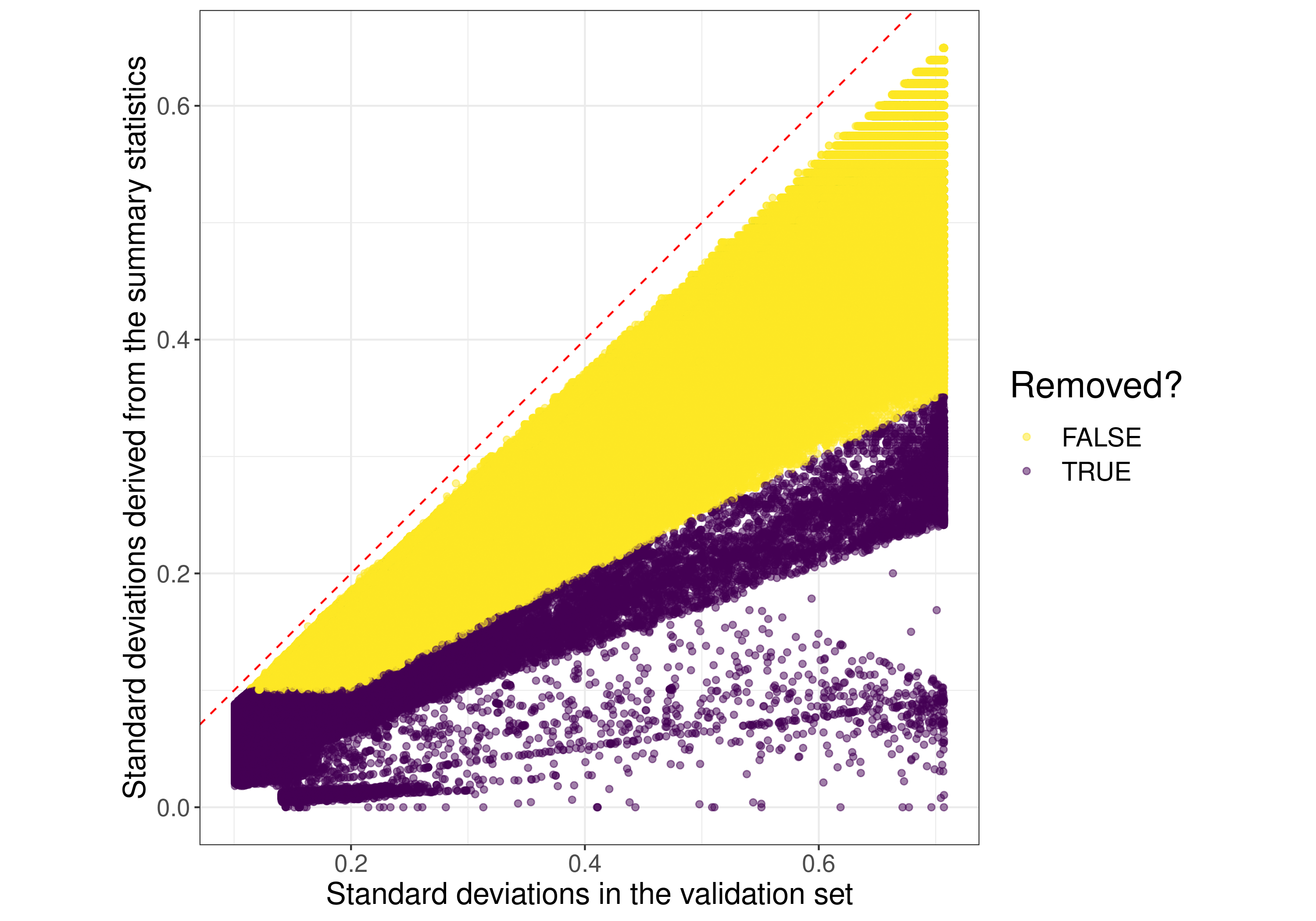

Supplement: btab456_Supplementary_Data [file btab456_supplementary_data.zip › FigS3_BRCA.png]

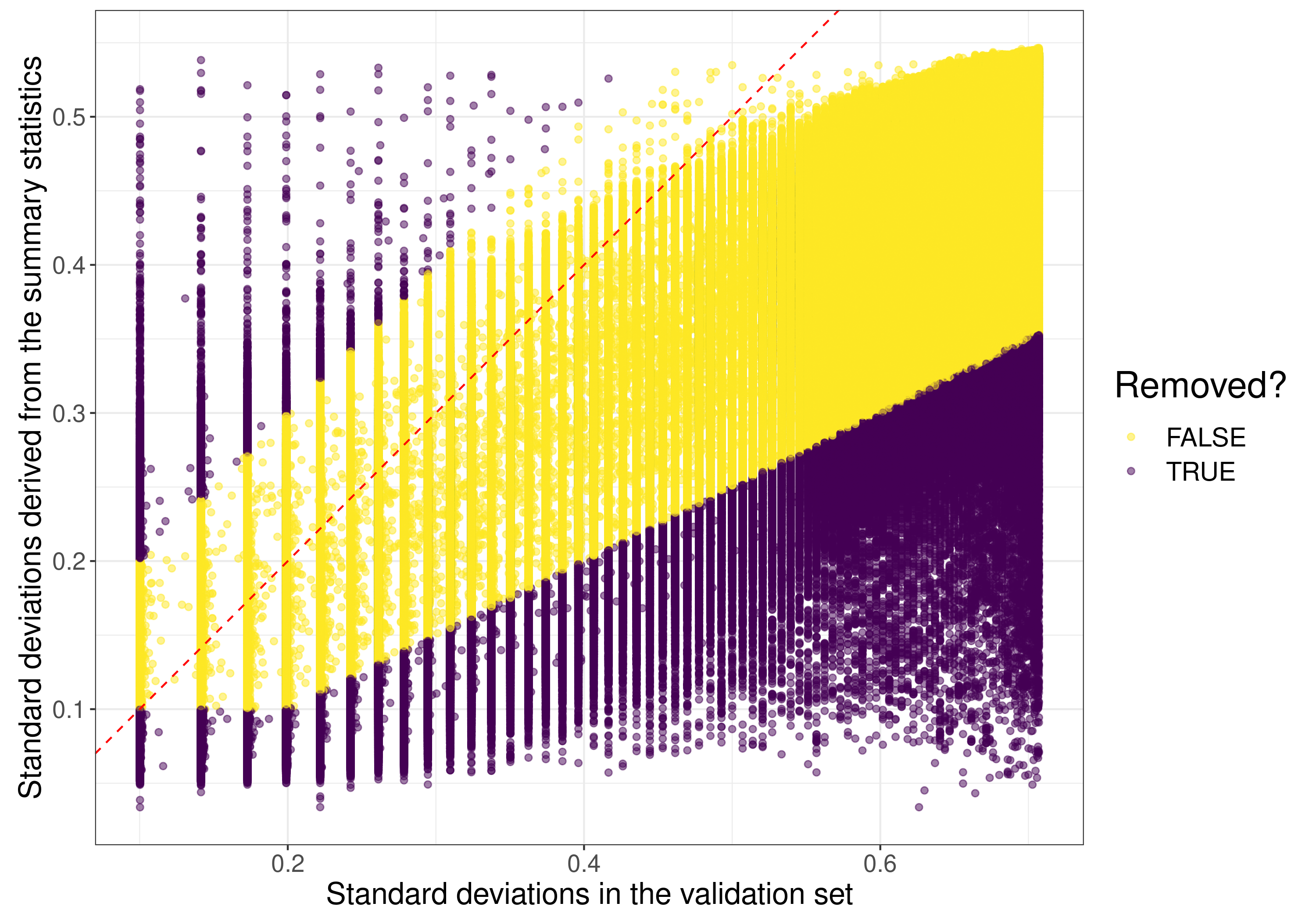

Supplement: btab456_Supplementary_Data [file btab456_supplementary_data.zip › FigS4_CAD.png]

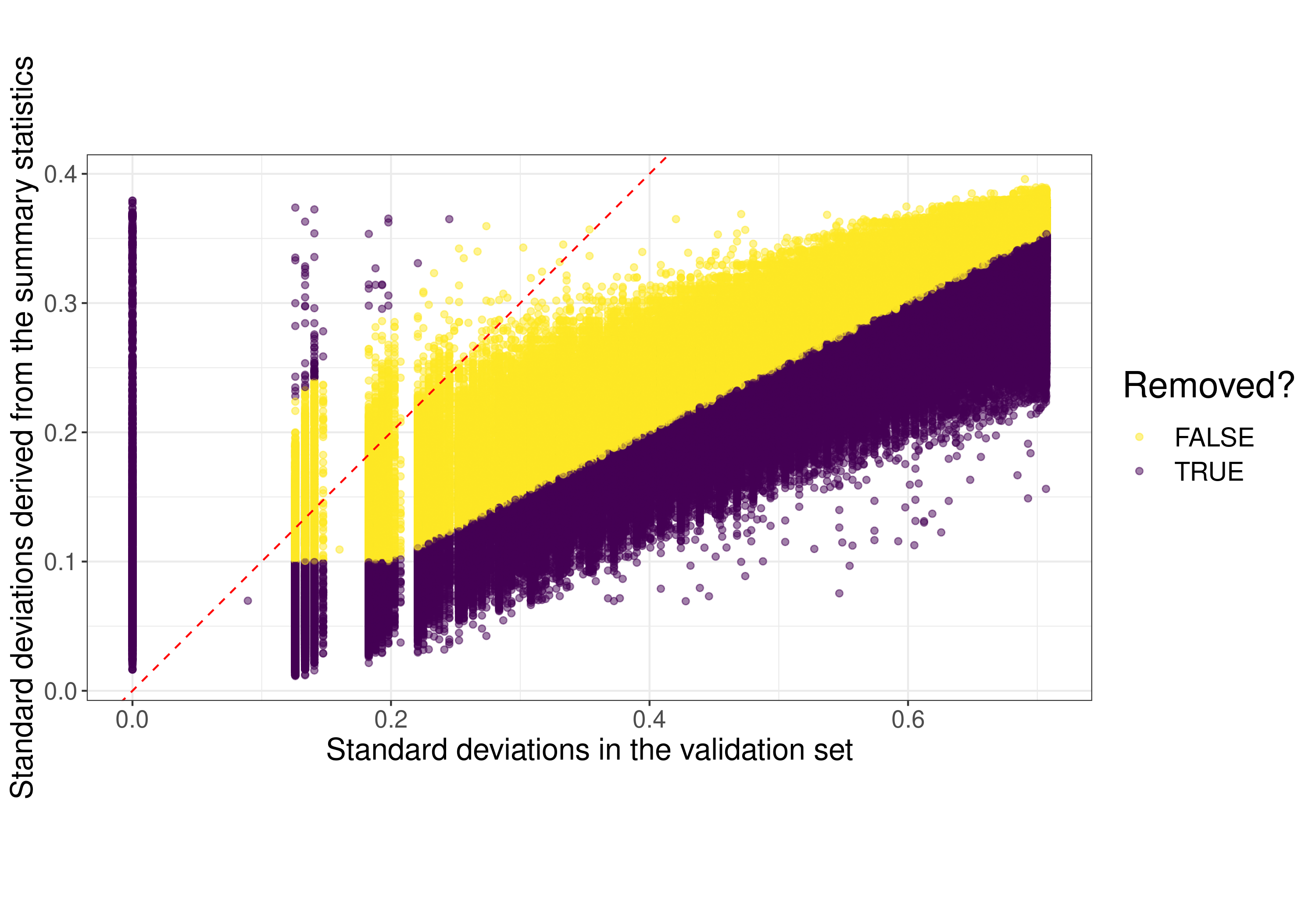

Supplement: btab456_Supplementary_Data [file btab456_supplementary_data.zip › FigS5_HEIGHT.png]

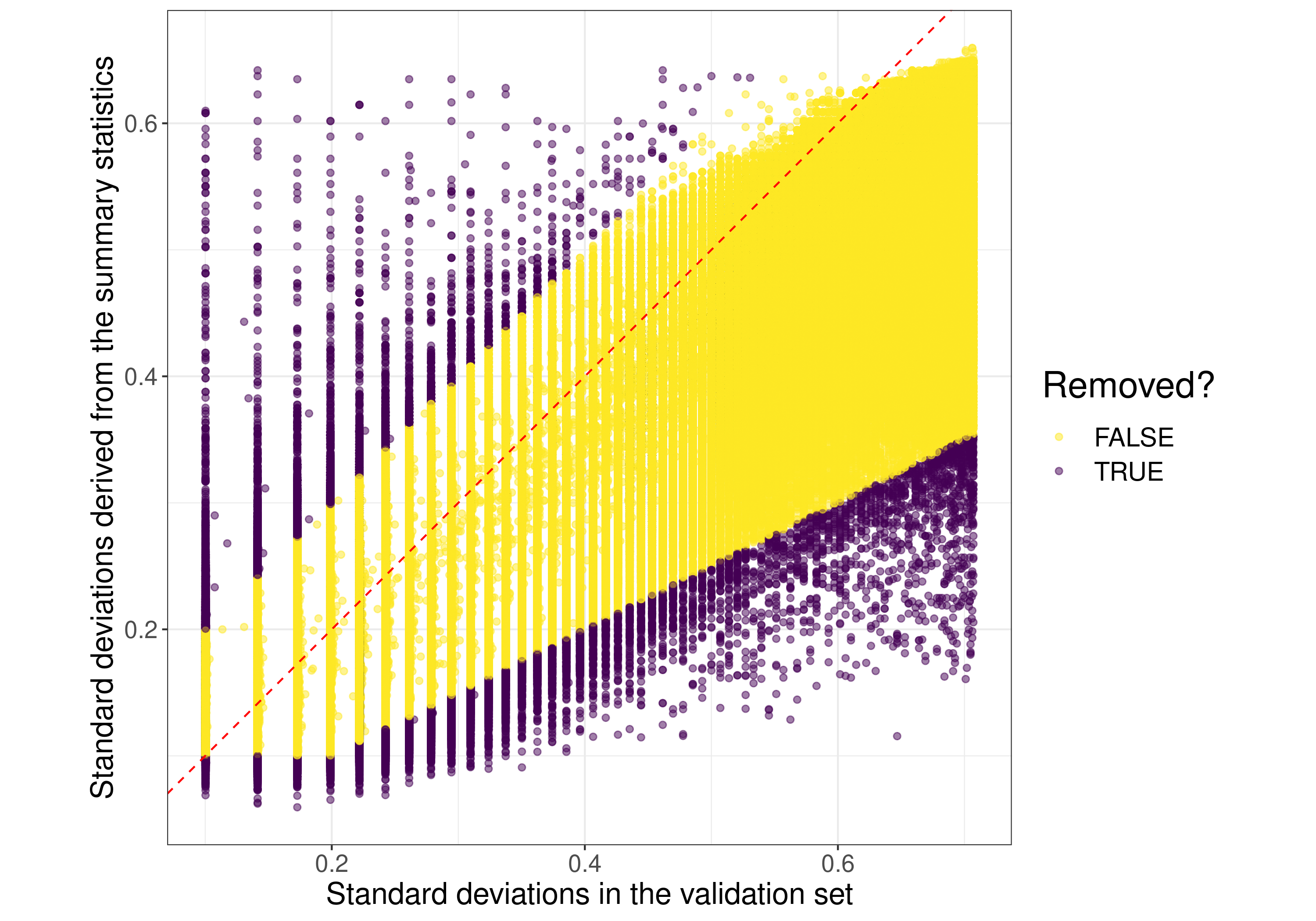

Supplement: btab456_Supplementary_Data [file btab456_supplementary_data.zip › FigS6_MDD.png]

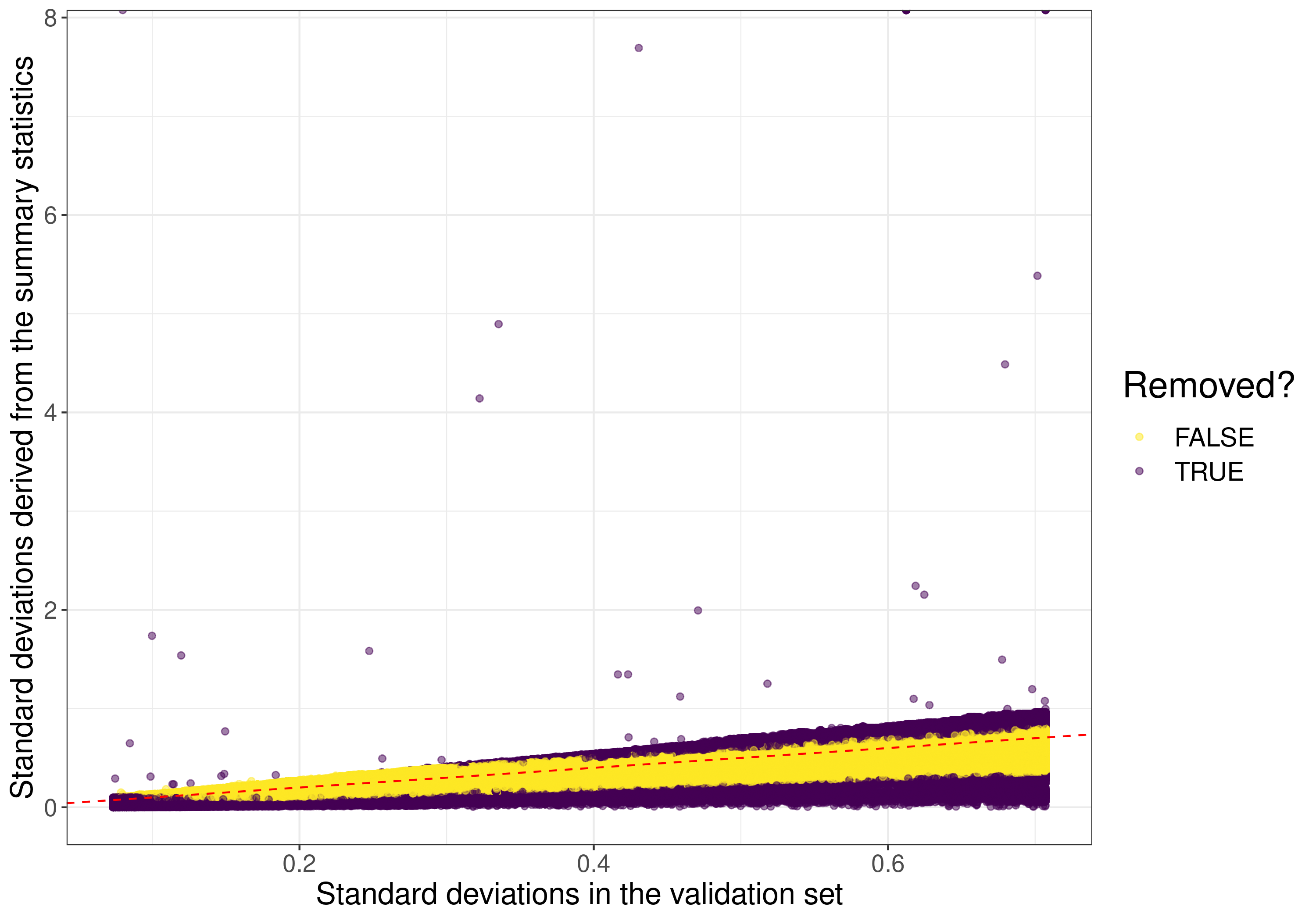

Supplement: btab456_Supplementary_Data [file btab456_supplementary_data.zip › FigS7_PRCA.png]

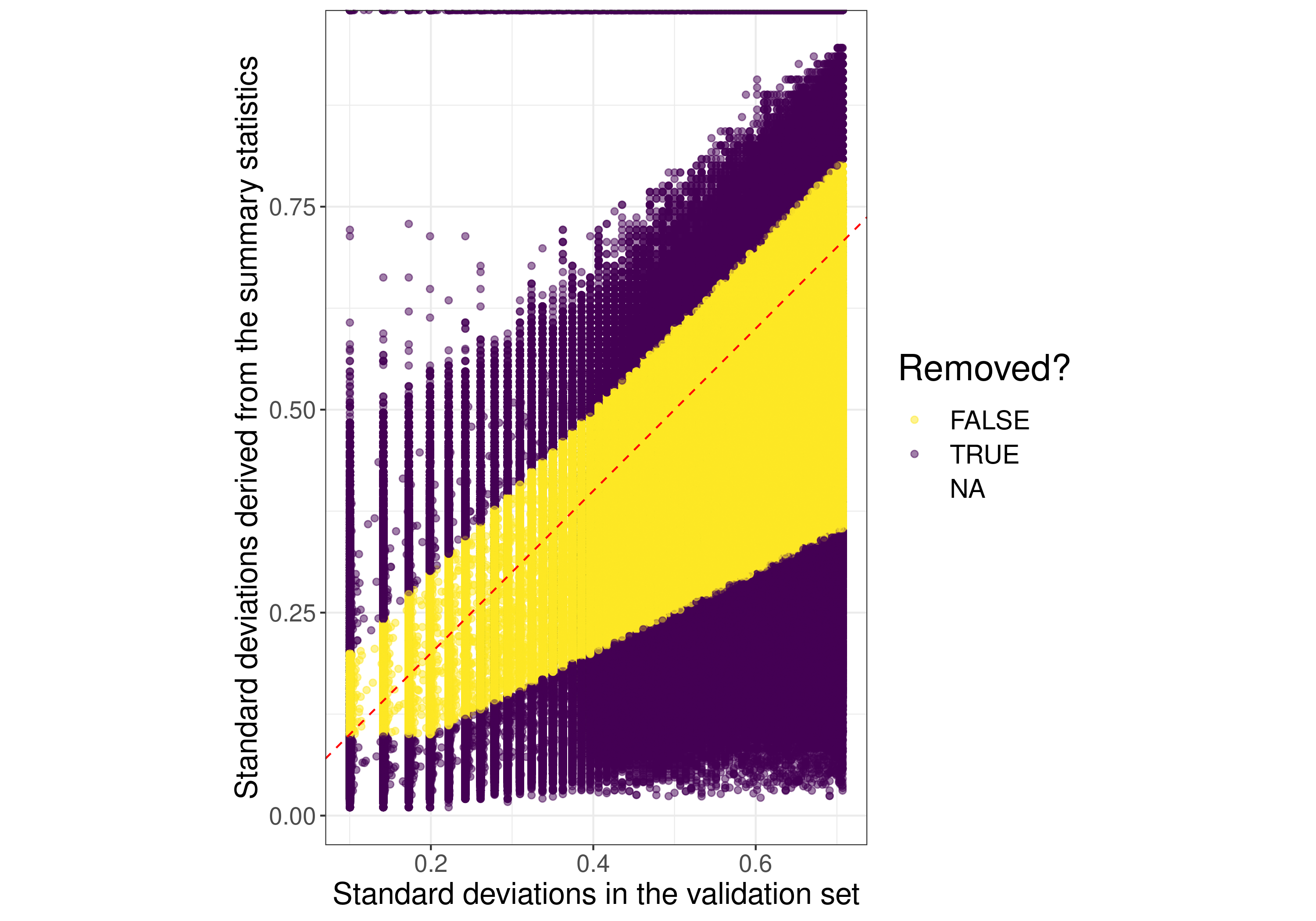

Supplement: btab456_Supplementary_Data [file btab456_supplementary_data.zip › FigS8_RA.png]

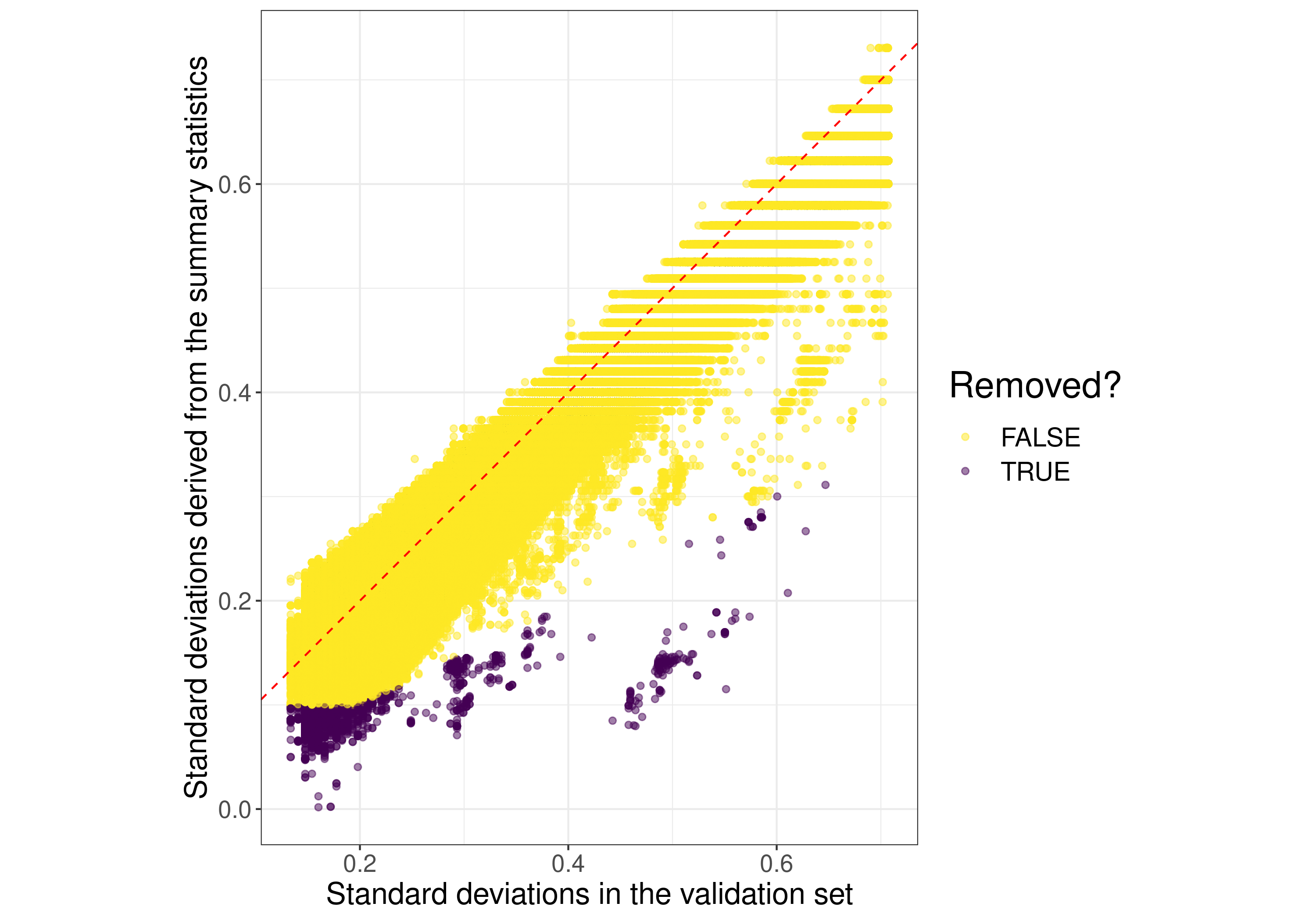

Supplement: btab456_Supplementary_Data [file btab456_supplementary_data.zip › FigS9_T1D.png]
